# Heterologous Expression of Wheat VERNALIZATION 2 (TaVRN2) Gene in *Arabidopsis* Delays Flowering and Enhances Freezing Tolerance

**DOI:** 10.1371/journal.pone.0008690

**Published:** 2010-01-13

**Authors:** Amadou Diallo, Ndjido Kane, Zahra Agharbaoui, Mohamed Badawi, Fathey Sarhan

**Affiliations:** Département des Sciences biologiques, Université du Québec à Montréal, Succursale Centre-ville, Montréal, Québec, Canada; University of Massachusetts Amherst, United States of America

## Abstract

The vernalization gene 2 (*VRN2*), is a major flowering repressor in temperate cereals that is regulated by low temperature and photoperiod. Here we show that the gene from *Triticum aestivum* (*TaVRN2*) is also regulated by salt, heat shock, dehydration, wounding and abscissic acid. Promoter analysis indicates that *TaVRN2* regulatory region possesses all the specific responsive elements to these stresses. This suggests pleiotropic effects of TaVRN2 in wheat development and adaptability to the environment. To test if TaVRN2 can act as a flowering repressor in species different from the temperate cereals, the gene was ectopically expressed in the model plant *Arabidopsis*. Transgenic plants showed no alteration in morphology, but their flowering time was significantly delayed compared to controls plants, indicating that *TaVRN2*, although having no ortholog in *Brassicaceae*, can act as a flowering repressor in these species. To identify the possible mechanism by which *TaVRN2* gene delays flowering in *Arabidopsis*, the expression level of several genes involved in flowering time regulation was determined. The analysis indicates that the late flowering of the 35S::*TaVRN2* plants was associated with a complex pattern of expression of the major flowering control genes, *FCA*, *FLC*, *FT*, *FVE* and *SOC1*. This suggests that heterologous expression of *TaVRN2* in *Arabidopsis* can delay flowering by modulating several floral inductive pathways. Furthermore, transgenic plants showed higher freezing tolerance, likely due to the accumulation of *CBF2*, *CBF3* and the *COR* genes. Overall, our data suggests that *TaVRN2* gene could modulate a common regulator of the two interacting pathways that regulate flowering time and the induction of cold tolerance. The results also demonstrate that *TaVRN2* could be used to manipulate flowering time and improve cold tolerance in other species.

## Introduction

In temperate regions, low temperature (LT) constitutes a major factor that regulates flowering time and many developmental transitions such as germination, bud dormancy and bursting [1]. In response to LT-conditions, plants cold acclimate and vernalize to prevent the sensitive floral meristem from freezing damages during the winter by postponing flowering [2]. The ability of plants to switch from vegetative to reproductive phase after a long period of cold, a process known as vernalization [3], allows plants to promote flowering early in the spring. During this cold exposure period, LT-responsive genes *CBFs* (*C-repeat binding factor*) and *COR* (*Cold Regulated*) are activated, allowing plants to increase their tolerance to cold and survive the winter ([4]). Cereals are classified into spring and winter growth habit according to their vernalization requirement [5], [6]. Spring varieties do not respond to vernalization and flower rapidly whereas winter varieties have a quantitative vernalization requirement. Therefore, winter varieties require vernalization to accelerate flowering and complete their life cycle. Understanding the genetic/molecular basis of both LT-responsive pathways (cold acclimation and vernalization), can help to better manipulate, the two important agronomical traits flowering and freezing tolerance.

In *Arabidopsis thaliana*, two key loci *FRIGIDA* (*FRI*) and *FLOWERING LOCUS C* (*FLC*) determine the difference in the flowering time and freezing tolerance [7], [8]. Spring ecotypes such as *Landsberg erecta* and *Columbia* have a non-functional *FRI* and/or a weak allele of *FLC* whereas winter annuals such as *Stokholm* and *San Felieu2* have functional *FRI* and *FLC* genes [5], [9]). *FLC*, a MADS-box gene, is the central regulator of flowering through the vernalization pathway. A high level of FLC expression (RNA and protein) induces a very late-flowering phenotype in winter annual ecotypes. In contrast, repression of FLC by vernalization causes an early-flowering phenotype [10], [11]. A recent study showed that FRI, which encodes a novel nuclear protein with no conserved domains except for two coiled coil regions, delays flowering in *Arabidopsis* through a co-transcriptional mechanism involving direct interaction with the nuclear cap binding complex, with concomitant effects on FLC mRNA splicing and transcription [12].

In cereals, vernalization and cold acclimation regulatory gene networks are also interconnected but are regulated by different factors. It was hypothesized that the temperate cereals have evolved the ability to use the presence of *VRN1* in the leaves as a signal to down regulate the *COR* genes [13]. This hypothesis does not necessarily imply a direct interaction between *VRN1* and *CBF* or *COR* genes [14]. However, the molecular and genetic mechanisms involved in the association between the up regulation of VRN1 and the down regulation of COR genes are not yet determined. Genetic analysis and expression profiling studies in wheat showed that *VRN1* transcript accumulation is associated with the vernalization response and the transition from the vegetative to the reproductive phase [13], [15], [16]. *VRN1* encodes a FRUITFULL-like MADS-box protein that belongs to the AP1/SQUA-like clade of transcriptional regulators whose members have been implicated in meristem identity and flower development [17], [18]. VRN2 is a zinc finger transcription factor that acts as a dominant negative regulator of flowering time in the vernalization pathway in wheat and barley [19]–[21]. Reducing *VRN2* level by RNAi accelerates flowering in hexaploid wheat [21]. Thus, there are obvious differences in the flowering regulation pathway between *Arabidopsis* and cereals. No genes corresponding to *FLC* were found in monocots ([22], [23]) as well no TaVRN2 orthologs were detected in *Brassicaceae* [21]. These observations suggest that monocots and dicots have separately evolved their vernalization pathway recruiting different components for analogous functions. The reciprocal control of flowering by the rice *SOC1* gene in transgenic *Arabidopsis* and by the *Arabidopsis FLC* gene in transgenic rice [24] was an indication that the flowering genes from one species could be used to manipulate flowering time in other species. In support of this hypothesis we showed that overexpressing the wheat MADS-box genes *TaVRN1* or *TaVRT2* modulates flowering time in *Arabidopsis* [25]. This cross species function of flowering genes may help to understand the evolution of the molecular mechanisms underlying vernalization and flowering time in plants.

In the present study, we provide further evidence of the cross species function of flowering genes. We showed that the wheat vernalization gene *TaVRN2* can act as a flowering repressor outside the cereal group. *Arabidopsis* transgenic plants expressing *TaVRN2* showed a late-flowering phenotype and enhanced freezing tolerance without any alteration in their morphology. These modifications are associated with changes in the expression of the flowering and freezing tolerance responsive genes.

## Materials and Methods

### Plant Material and Growth Conditions

Two wheat varieties (*Triticum aestivum*, 2n×6 = 42), a spring habit cultivar Manitou and a winter habit cultivar Norstar, were grown in a controlled growth chamber as previously described [13]. Briefly, plants were grown in a growth chamber at 20°C under long days (LD) (16 h at 320 µmol m^−2^ s^−1^) or short days (SD) (8 h at 320 µmol m^−2^ s^−1^) conditions. For vernalization treatment, 14 days old plants were grown at 4°C for 0, 7, 14, 21, 28, 35, 42, 49, 56, 77 and 98 days under either SD and LD.

For abiotic stress treatment, 7 days old wheat plants were treated as follows: for cold, seedlings were cold treated for one day at 4°C (CA); for heat shock, they were exposed at 40°C for 1 and 3 h; wounding stress was induced by cutting seedlings into 1 cm segments and placing them in water at 20°C for 3 and 14 h; salt-stressed plants were obtained by incubating seedlings for 18 h with 300 or 500 mM NaCl; water stress was induced by removing seedlings from vermiculite and leaving them at 20°C without water for different time periods, after which the relative water content (RWC) was evaluated; for ABA treatment, 100 mM ABA in 0.02% (v/v) Tween-20 for 18 h were sprayed on seedlings.

For genes expression, flowering time (leaf number) and freezing experiments in *Arabidopsis thaliana*, seeds were put on soil and kept in a cold room (4°C) in the dark for 2 days. After this treatment, seeds were transferred to a growth chamber at 20°C under long days (16 h at 120 µmol m^−2^ s^−1^) conditions. For the cold treatment, 15 days old plants grown at 20°C under long day were transferred to 4°C for four weeks. Flowering time was measured as the number of rosette leaves every week.

### Cloning of *TaVRN2* genes and its promoters (supplementary material S2)

One homologous copy of the *VRN2* gene in hexaploid wheat (*TaVRN-B2*) was isolated from wheat cDNAs libraries as previously described [26]. The second copy, *TaVRN-A2*, was PCR-amplified from genomic DNA (*Triticum aestivum* L.) using specific primers (**Table S1**). Promoters of *VRN2* genes from spring and winter wheat were also PCR-amplified from genomic DNA using specific primers (**Table S1& Sequences S1**).

### Chromosome localization of *TaVRN-2 genes*

Genomic DNA was extracted from several stocks of the wheat cultivar Chinese Spring: ditelocentric series provided by the USDA from E. R. Sears collection.

From the diluted genomic stocks, 2 µL (20 ng) was used as a template in a 25 µL.

PCR reaction containing 1 X TaqMan universal PCR master mix (Invitrogen), 0.9 µM non-fluorescent primers, and 0.25 µM TaqMan-MGB fluorescent probe. The PCR thermal cycling parameters were 50°C for 2 min, 95°C for 10 min followed by 45 cycles of 95°C for 15 s and 60°C for 1 min. At the end of the run, the Ct values were compared, and genetic stocks that showed a delayed or undetectable amplification were identified as the location of the assayed *TaVRN-2* gene.

### Overexpression of *TaVRN2* in *Arabidopsis*

*Agrobacterium tumefaciens* carrying the binary vector *pGreenII0029/35S::TaVRN-B2* was used to transform *Arabidopsis (Col 0)* using the floral dip method (Clough and Bent, 1998). Wild type and plants transformed with *pBIN19/35S::GUS* were used as controls. T_1_ seeds were selected on 0.5x MS salts and 1x MS vitamins containing 50 µg ml^–1^ kanamycin. Transgenic T_1_ seedlings were transferred to soil and kept at 20°C day/night in a growth chamber and grown to maturity under LD conditions (16 h photoperiod) at 20°C (day/night) to produce T2 and T3 lines as previously described [25]. Three independent homozygous lines of the fourth generation were used for the experiments.

### Determination of freezing tolerance

A Caltec Scientific Ltd. Model 8-792 Large Capacity Temperature Stress Chamber was used to perform the FT tests. This instrument consists of four major component systems: a Sanyo Model MDF-792 24.75 ft^3^ capacity ultra-low temperature chest freezer, a custom designed stainless steel plenum box with its integral blower and heater (provides air circulation and heating) and an Omega Engineering Inc. Model CN3002 programmable profile controller (monitors the test-chamber air temperature). The controlled action of the heater combines with the constant cooling of the freezer to achieve the desired temperature at any given time.

Non-acclimated (NA) and cold-acclimated (CA) plants were grown in soil for 3 weeks and subjected to the following freezing treatment. The temperature was lowered gradually to –6.5°C for NA and to –10.5°C for CA plants (2°C h^–1^) and maintained at this temperature for 6 h. The temperature was then gradually increased to 4°C. To determine temperature variability in the freezer, temperatures were monitored by four independent thermocouples T probes distributed in the freezer and connected to an Agilent 3497-0A data acquisition/switch unit. Freezing regimes that showed more than 0.5°C discrepancies between the different probes were rejected. To minimize light stress effect after the freezing treatment, plants were thawed at 4°C for 24 h in the dark and then moved to growth chamber (20°C) under low light for an additional 24 h before returning to normal light conditions. Representative pictures were taken 2 weeks after the freezing test. Eighteen plants were frozen per line per assay, and the experiment was repeated at least three times.

### Gene expression studies in wheat

Total RNAs were isolated from wheat plants as described previously [27], reverse-transcribed, and subjected to quantitative real-time PCR on an ABI PRISM 7000 Sequence Detection System (Applied Biosystems). The PCR thermal-cycling parameters were 50°C for 2 min, 95°C for 2 min, followed by 50 cycles of 95°C for 20 sec and 60°C for 1 min. For qRT-PCR, relative transcript abundance was calculated and normalized with respect to 18S ribosomal RNA transcript levels. Data shown represent mean values obtained from four independent amplification reactions, and the error bars indicate the ±SE of the mean. Each experiment was repeated three times. TaqMan primers set were designed according to the specific sequence of the target gene (Invitrogen) (Table S1). All calculations and analyses were performed using SDS RQ Manager 1.1 software using the 2^−ΔΔ^Ct method with a relative quantification (RQ)min/RQmax confidence set at 95% [28]. The error bars display the calculated maximum (RQmax) and minimum (RQmin) expression levels that represent SE of the mean expression level (RQ value). The upper and lower limits define the region of expression within which the true expression level value is likely to occur (SDS RQ Manager 1.1 software user manual; Applied Biosystems). Amplification efficiency (98% to 100%) for the two primer sets was determined by amplification of cDNA dilution series using 80, 20, 10, 5, 2.5, and 1.25 ng per reaction (data not shown). Specificity of the RT-PCR products was assessed by gel electrophoresis. A single product with the expected length was obtained for each reaction.

### Gene expression in Arabidopsis

Total RNA was extracted from leaves harvested in the middle of the day and transcript level of flowering genes was measured by qRT-PCR using Syber Green. The PCR thermal-cycling parameters were 94°C for 2 min, followed by 40 cycles of 94°C for 20 sec and 58°C for 1 min and an extension of 72°C for 10 min. Each value is the mean of four separate qPCR reaction normalized to *ACTIN2*. Each experiment was repeated two times using RNA prepared from two biological samples with similar results.

For *AtCBFs* and *AtCORs* genes expression analysis, total RNAs were isolated from the leaves of *Arabidopsis* plants at different stages as described for each experiment. RT-PCR analyses were performed using SuperScript™ First-Strand Synthesis System for RT-PCR KIT according to instructions (Invitrogen).

Specific probes of flowering and cold associated genes used are presented in Table S1.

All the experiments were repeated at least three times with three biological replicas.

### Statistical Analysis

Results were expressed as mean_SEM of three experimental repeats using different plants. Comparison between groups and analysis for differences between means of control and treated groups were performed using ANOVA followed by the post-hoc test Newman–Keuls (P<0.05). The threshold for statistical significance was: *: P<0.05, **: P<0.01, ***: P<0.001 and ns: P>0.05.

## Results

### Structural analysis of *VRN2 in T. aestivum*

Two *TaVRN2* sequences were cloned from *T. aestivum* cv Norstar. The first sequence of 642 bp length was PCR-amplified from wheat cDNA libraries and the second copy of 621 bp length was amplified from genomic DNA (Sequences S1). Alignment at the nucleotide level showed 97% identity between the two sequences with deletion of 21 bp in the first exon and several single nucleotide polymorphisms (SNPs). BLAST search at the DNA level revealed that the expressed sequence cloned from the cDNA libraries shared 96% identity with *TmVRN2* (AY485963.1) from the *T. monoccocum* that carries the diploid genome (AA). This relative low identity suggests that the 642 bp sequence is not the copy A. However, it shows a very good score with the Zinc finger-CCT domain gene (*ZCCT1-B1*) of *Triticum turgidum* ssp. dicoccon; AB genome. A homology search also shows that the 621 bp sequence has 99% identity with the A genome of *TdVRN2* (AY485979.1) indicating that the 621 bp could be assigned as the copy A of *TaVRN2*. Using genomic DNA extracted from ditelocentric wheat lines, the 621 bp sequence was mapped on chromosome 5A whereas the 642 bp sequence was mapped on chromosome 4B as recently reported [29]. Together with the DNA homology, the data indicate that the 642 bp sequence represents the copy B of *TaVRN2* gene (so-called *TaVRN-B2*) and the 621 bp sequence represents the copy A of the gene (*TaVRN-A*2).

**Figure 1:**
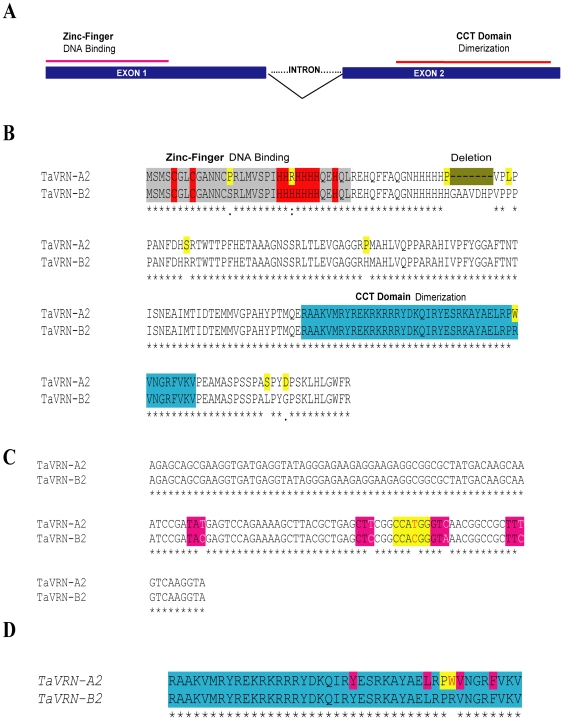
*TaVRN2* structure analysis. A) Gene structure showing the two exons and one intron. Exon1 contains the zinc finger domain and exon2 contains the CCT domain. B) The alignment of TaVRN-A2 and TaVRN-B2 proteins. Regions of putative zinc finger highlighted in red, CCT domains highlighted in blue and difference of nine amino acids highlighted in yellow. C) The alignment of CCT domain of the two copies of *TaVRN2* showing their different SNPs (in white and red). The sequence highlighted in yellow in the *TaVRN-A2* corresponds to NcoI restriction site. D) Proteins alignment of CCT domain, Alignments are done by using Clustal W program from the web.

At the protein level, TaVRN-A2 has a seven amino acid deletion (His 49-His 55) compared to TaVRN-B2 (Fig. 1B). Structural analysis of these two proteins indicated the presence of a zinc-finger DNA binding domain located in the first exon and a CCT (CONSTANS, CONSTANS-like and TOC) dimerization domain in the second exon (Fig. 1A). The protein alignment of the two copies is shown in figure 1B. The alignment of the CCT domain of TaVRN-A2 and TaVRN-B2 shows four silent polymorphisms and a substitution of a cytosine by a thymine base (Fig. 1C and 1D). This polymorphism changes the amino acid codon at position 35 of the CCT domain from arginine to tryptophan (Fig. 1C).

### *TaVRN2* homeologous genes show differential expression profile

**Figure 2:**
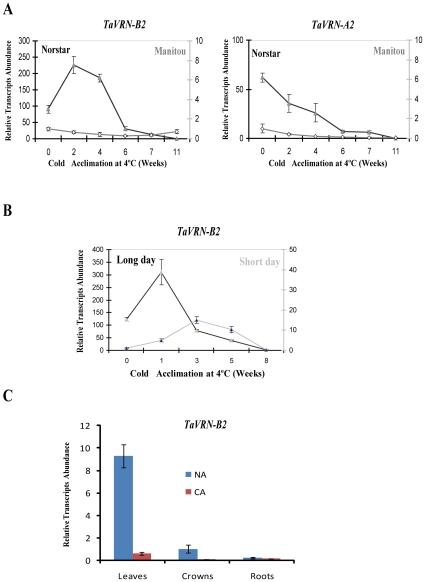
Transcripts level of *TaVRN-B2 and TaVRN-A2* in both spring and winter wheat during cold acclimation. A) *TaVRN-B2 and TaVRN-A2*, in cold acclimated plants. Wheat plants were grown for 14 days at 20°C under a long day (16 h) photoperiod, transferred to 4°C under identical photoperiods, and then sampled at regular intervals. B) *TaVRN-B2* in cold acclimated plants under short day and long day conditions. Winter wheat plants were grown for 14 days at 20°C under either a long day (16 h) or a short day (8 h) photoperiod, transferred to 4°C under identical photoperiods, and then sampled at regular intervals. qRT-PCR were done using total reverse-transcribed RNA isolated from wheat aerial part. C) *TaVRN-B2* relative transcripts abundance in different tissues. Winter wheat were grown for 7 days at 20°C. Non-acclimated control plants (NA) were maintained at 20°C for 6 days. Cold-acclimated plants (CA) were transferred at 4°C for 36 days. Total RNA was isolated from leaves, crown and roots, reverse-transcribed and subjected to qRT-PCR. Data shown represent mean values obtained from independent amplification reactions (n = 4), and the error bars indicate the range of possible RQ values define by the SE of the delta threshold cycles (Cts). Experiment was repeated three times with similar results.

To determine *TaVRN2* expression level, total RNAs were extracted from different parts of the winter wheat seedlings (leaves, crowns and roots) and reverse-transcribed for qRT-PCR analyses. The data indicated that *TaVRN2* transcripts accumulated mostly in leaves, lesser in crown and was barely detectable in roots. The highest level of accumulation is found in 6 days old leaves from non acclimated plants. In LT-acclimated winter wheat, the level of *TaVRN2* transcripts in leaves declined to reach the lowest level after 6 weeks (Fig. 2A). *In situ* hybridization experiments showed that *TaVRN2* transcripts are detected in apices cells of winter wheat during the vegetative state [26]. Together, these data suggest that the LT signal modulating the expression of *TaVRN2* genes could be perceived by either the leaves or the apices.

To test if *TaVRN2* homeologous genes show differential expression patterns, qRT-PCR were performed using specific primers designed to preferentially amplify the A and B copies. The results indicated that the transcripts of the two homologous genes are regulated by LT exposure in the winter genotype (Fig. 2). The transcripts level of the two copies remains high during the early stage of LT exposure and starts to decline toward the vernalization saturation point, where the low temperature treatment no longer reduce the final leaf number (FLN). However, *TaVRN-A2* transcript accumulates to a lesser level compared to *TaVRN-B2* (Fig. 2A). The down-regulation of *TaVRN2* transcripts by vernalization is associated with the up-regulation of *TaVRT-1* also called *TaVRN-1* as shown by our previous reports [26]. The results also showed that the two copies of *TaVRN2* transcripts accumulate to a higher level in winter wheat compared to spring wheat during cold acclimation (Fig. 2A). In addition, *TaVRN-B2* accumulates to higher level during the first three weeks of cold acclimation in winter wheat grown at LD compared to SD. (Fig. 2B). This result is in agreement with previous data in barley [30] that showed vernalization has a greater effect on *HvVRN1* than *HvVRN2* while photoperiod has higher effect on *HvVRN2* than *HvVRN1*. It was also shown that in some genotypes of wheat, the vernalization requirement could be replaced by 6 weeks of short day treatment [31].

### *TaVRN2* is regulated by various stresses

**Figure 3:**
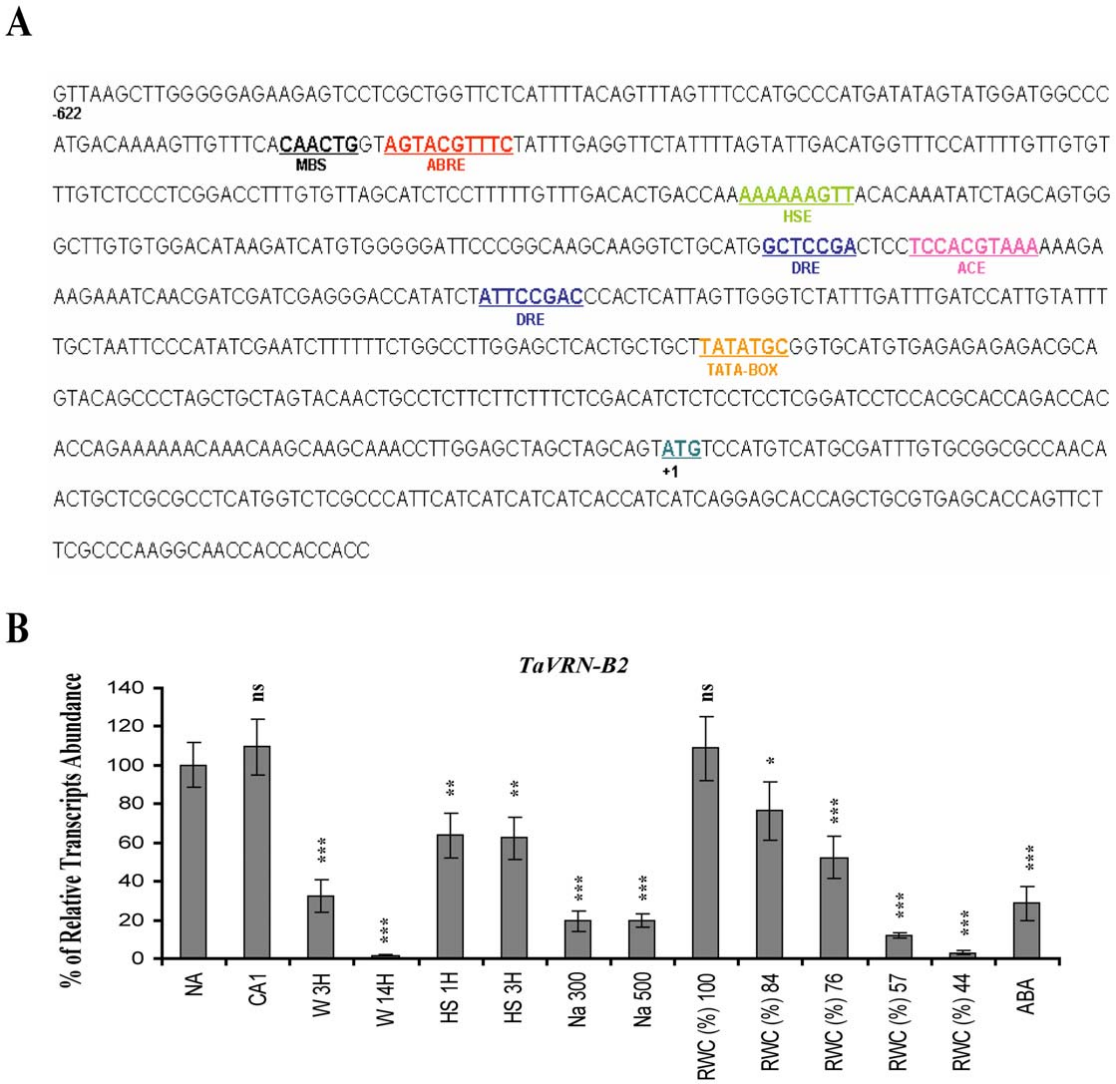
Promoter analysis and transcripts level of *TaVRN2* in response to various abiotic stresses. A) Putative regulatory *cis*-elements in *TaVRN2* promoter (**MBS:** MYB binding site involved in drought-inductibilty, **ABRE:** *cis*-acting element involved in abscisic acid responsiveness, **HSE:** *cis*-acting element involved in heat stress responsiveness, **ACE:** cis-acting element involved in light responsiveness, **DRE:** cis-acting element involved in cold stress and TATA-box). The plant CARE and PLACE programs were used for the promoter analysis. B) Transcripts abundance in winter wheat exposed to various stresses. Seven days old plants were exposed to different stresses: Non-acclimated (NA), cold acclimated (CA), wounding (W), heat shock (HS), salt (NaCl), water stress (RWC), abscisic acid (ABA). Data shown represent mean values obtained from independent amplification reactions (n = 4), experiment was repeated three times with similar results. A statistical difference between each sample and the expression observed in non acclimated plants is indicated by an asterisk on top of each histogram columns. The threshold for statistical significance was: *: p<0.05; **: p<0.01; ***: p<0.001; ns: P>0.05.

Sequence analysis of *TaVRN2* promoter region revealed the presence of several putative *cis*-elements involved in cold stress, heat shock, light sensitivity, water stress, wounding and abscissic acid responsiveness (Fig. 3A & Sequences S1). These motifs, except ABRE, are conserved *in T. monoccocum* while barley promoter has only the MBS and DRE. To test the functionality of these motifs, the expression of *TaVRN2* genes was monitored in winter wheat leaves exposed to various abiotic stresses (see the experimental procedures). The results revealed that, *TaVRN-B2* transcripts are repressed by abscissic acid, water stress, heat shock, salt, and wounding treatments (Fig. 3B). The biological significance of the stress regulation of *TaVRN2* requires further investigation.

### TaVRN2 acts as a flowering repressor in *Arabidopsis*

**Figure 4:**
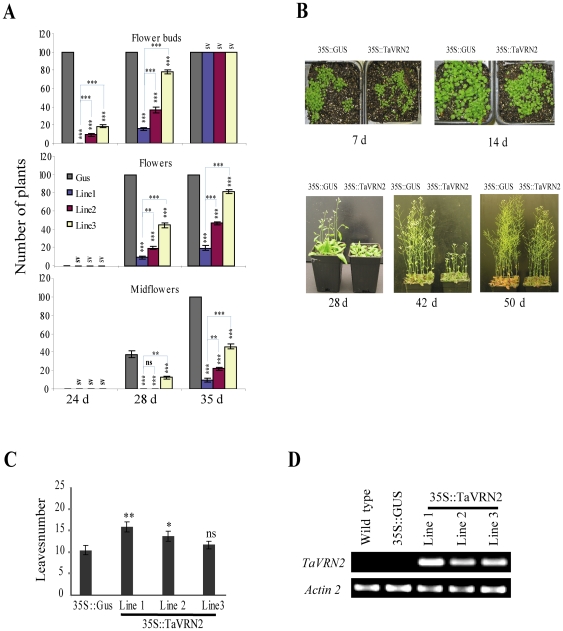
Analysis of transgenic *Arabidopsis* plants overexpressing *TaVRN-B2*. A) Effect of *TaVRN-B2* expression on flower initiation and development during growth under long day conditions at 20°C. The number of plants is expressed as means SEM (n = 99 plants). A statistical difference measurement from three independent experiments. The threshold for statistical significance is indicated by an asterisk on top of each histogram columns; in vertical, comparison between GUS and the three LINES and in horizontal, comparison between Line1 and two others lines; *: P<0.05, **: P<0.01, ***: P<0.001, ns: P>0.05 and sv: same value, statistical analysis is not possible. B) Phenotypic effect of TaVRN-B2 overexpression in *Arabidopsis*. Control plants *35S::GUS* (GUS) and transgenic plants *35S::TaVRN-B2* (Line1) were grown under long day conditions at 20°C. Pictures were taken at 7, 14, 28, 42 and 50 days (d). C) Number of rosette leaves at the time of bolting in control plants (*35S::GUS*) and 3 transgenic lines *35S::TaVRN-B2* (L1–L3). Plants were grown under long-day conditions at 20°C and leaves were counted when the first bolt became visible. The number of leaves is expressed as means SEM (n = 18 plants). A statistical difference measurements from three independent experiments (n = 18). The threshold for statistical significance is indicated by an asterisk on top of each histogram columns; comparison between GUS and the three LINES; *: P<0.05, **: P<0.01, ***: P<0.001, ns: P>0.05. D) Transcript level of *TaVRN2* in controls (wild type (WT) and *35S::GUS*) and transgenic lines (L1–L3). Total RNA was extracted from leaves of 15 day-old plants grown under LD conditions at 20°C. *TaVRN-B2* transcript levels were measured by RT–PCR. Each experiment was repeated three times with three biological replicas.

To determine the function of TaVRN2 in modulating flowering time in *Arabidopsis*, transgenic plants expressing *TaVRN-B2* allele under the control of the CaMV 35S promoter (*35S::TaVRN2*) were compared with wild type *Arabidopsis* (Col-0) and transgenic plants expressing *GUS* under the control of the *CaMV 35S* promoter (*35S::GUS*). Among the *35S::TaVRN2* homozygous lines showing late flowering phenotypes under LD conditions, ten lines showing different levels of late flowering and transgene expression were randomly selected and brought to the next generation. These observations suggest that there is a correlation between phenotype and the transgene expression level. Based on the strength of the phenotype, seven of these lines were brought to the second generation for further analysis. Three independent lines that conserved the late flowering phenotypes were selected and brought to the third generation. Homozygous plants of the fourth generation were analysed (Fig. 4). The flowering time was measured by the number of rosettes of leaves when flowering occurs. The results show that this number was 10 leaves for control plants, and 15, 13 and 12 leaves for line1, line2 and line3 respectively (Fig. 4C). This flowering time was delayed by 7 days (P<0.01) in line 1 and 5 days in line 2 and 3 when grown under long day conditions confirming that the strength of delay of flowering was consistent with the transgene expression level (Fig. 4). Effect of *TaVRN-B2* expression on flowering initiation and development is shown in Fig. 4A. All control plants initiated flower buds and flower formation after 24 and 28 days respectively compared to the three transgenic lines. After 35 days the midflower formation is completed in control plants while the trangenic lines still at stage of flower formation. This demonstrates clearly, that *TaVRN-B2* overexpression delay flowering time and development in the dicot *Arabidopsis*.

**Figure 5:**
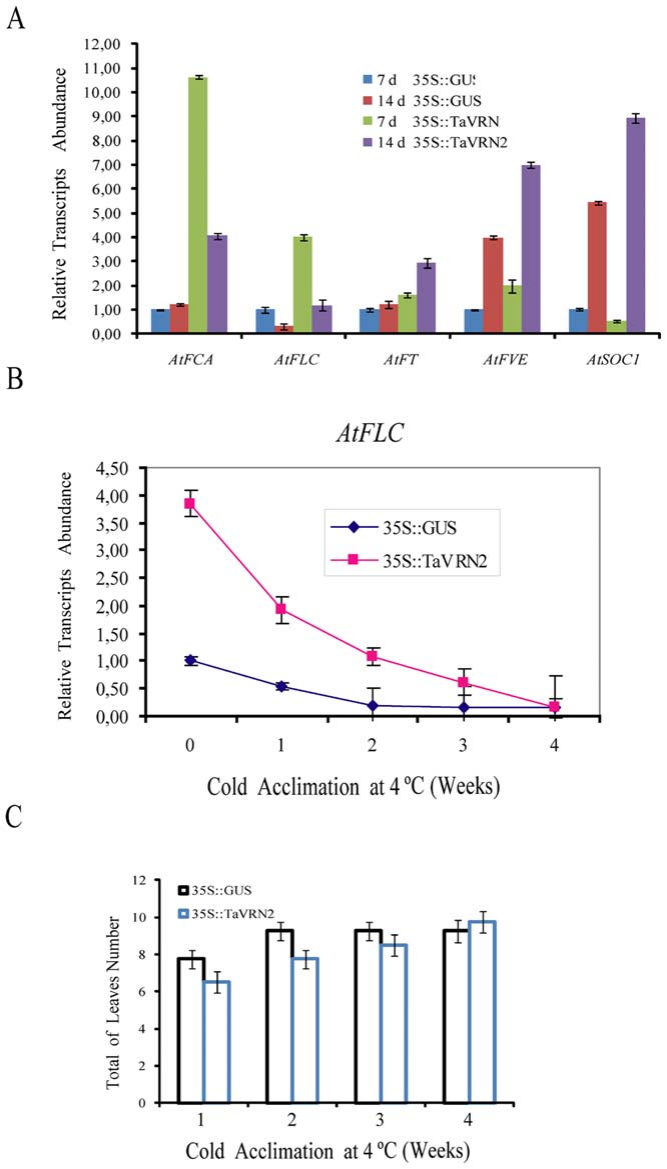
Effect of *TaVRN-B2* overexpression on *Arabidopsis* flowering genes. A) Expression pattern of flowering genes under unvernalized conditions in control (*35S::GUS*) and transgenic *35S::TaVRN-B2* plants. Seven and 14 days (d) old plants were grown at 20°C under LD conditions. Relative transcript abundance is normalized in relation to the level of each gene at 7 days of *35S::GUS* control sample. B) Expression pattern of *FLC* under cold acclimation conditions in control (*35S::GUS*) and transgenic *35S::TaVRN-B2* plants (Line 1). Fifteen days old plants were grown at 4°C for 5 weeks. Relative transcript abundance in normalized to the 35S::GUS control sample at zero time. C) Total number of leaves (n = 32) of cold acclimated control (*35S::GUS*) and transgenic *35S::TaVRN-B2* plants (Line 1) at bolting stage.

Since *VRN2* encoded a zinc-finger transcription factor [21], we assumed that its ectopic expression in *Arabidopsis* could delay flowering time through similar manner to *Arabidopsis* zing finger CO. To test this hypothesis, the effect of *TaVRN2* on genes involved in the flowering pathway was determined. Quantitative RT-PCR analyses of several flowering genes using RNA extracted from *35S::TaVRN-B2* (Line1) and control lines (*Col-0* and *35S::GUS*) grown under LD at 20°C were performed. The analysis (Figure 5A) showed an increase of *AtFLC* by 4 fold in the *35S::TaVRN-B2* transgenic plants compared to the control. This high expression of the central regulator *FLC* in the *35S::TaVRN-B2* plants during the vegetative phase suggests that *TaVRN2* may executes part of its action on *Arabidopsis* flowering time by acting on *AtFLC* transcription. On the other hand, the transcript of the autonomous pathway genes (*AtFCA* and *AtFVE*) and the flowering inducers (*AtFT* and *AtSOC1*) accumulate in transgenic plants, but this is not enough to induce flowering. We hypothesize that the level of accumulation of floral inducer genes is not sufficient to override the 4 fold increase of *FLC* transcript in transgenic plants.

To determine the effect of cold acclimation on FLC stability, two week plants were cold acclimated for four weeks. Quantitative RT-PCR analyses (Fig. 5B) showed that *AtFLC* transcripts level in *35S::TaVRN-B2* plants were higher during the cold acclimation period compared to controls plants. More interestingly, we observed that the control plants start bolting when the level of *AtFLC* decreased to the lower level after two weeks of cold treatment (Fig. 5C). The transgenic plants start bolting two weeks later concomitantly with the decline of *AtFLC* level (Fig. 5B and 5C). These results suggest that the high accumulation of *AtFLC* in the*35S::TaVRN-B2* plants are associated with the late flowering phenotypes.

### *TaVRN2* enhances freezing tolerance and up regulates *COR* genes expression in transgenic *Arabidopsis*

**Figure 6:**
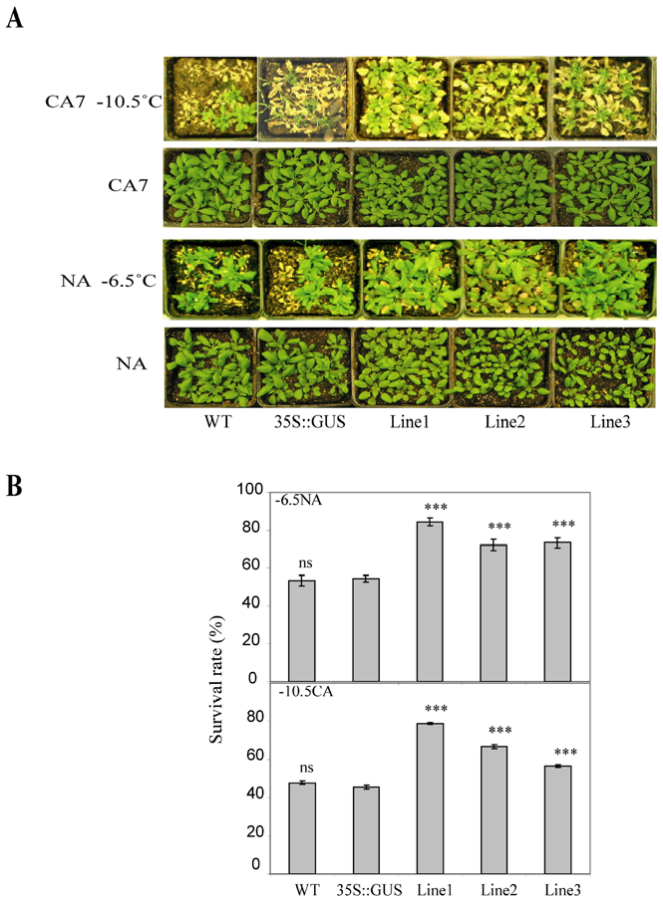
*TaVRN-B2* enhances freezing tolerance in *Arabidopsis*. A) Control and transgenic plants after freezing test. Plants were grown for 3 weeks at 20°C (NA) or grown for 3 weeks at 20°C then transferred to 4°C for 7 days (CA7). Controls plants wild type (WT) and *35S::GUS*), transgenic *35S::TaVRN-B2* lines (Line1–Line3). Plants were subjected to freezing test (NA frozen to −6.5°C and CA7 frozen to −10.5°C). Pictures were captured for the same plants before the freezing and after a recovery period of 2 weeks. B) Survival rate after freezing stress expressed as a percent of surviving plants. A statistical difference measurements from three independent experiments (n = 33). The threshold for statistical significance is indicated by an asterisk on top of each histogram columns; comparison between GUS and the three LINES; *: P<0.05, **: P<0.01, ns: P>0.05.

To test if the delay of flowering and the extension of the vernalization phase are associated with enhanced cold tolerance, freezing tests were performed on non-acclimated and acclimated *Arabidopsis* plants as described in the experimental procedures. Non-acclimated transgenic plants showed higher survival rate after freezing at −6.5°C compared to the control plants (Fig. 6). After cold acclimation the transgenic plants tolerated freezing of −10.5°C compared to cold acclimated controls (Fig. 6). These data indicated that *Arabidopsis* lines over expressing *TaVRN2* are more freezing tolerant than control lines.

**Figure 7:**
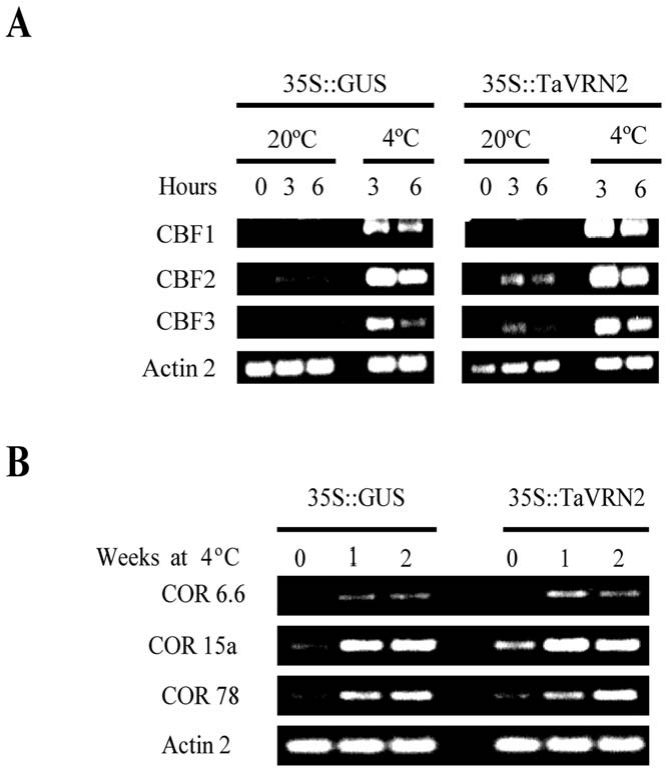
Effect of *TaVRN-B2* over-expression on the accumulation of cold-regulated transcripts. A) Transcript level of *CBFs* genes. Control (*35S::GUS*) and transgenic 35S::*TaVRN-B2* plants (line 1) grown under long day conditions and exposed to 4°C for 3 and 6 hours. B) Transcript level of *CORs* genes. Control (*35S::GUS*) and transgenic *35S::TaVRN-B2* plants (line 1) grown under long day conditions at 4°C for 1 and 2 weeks. Transcripts levels were measured by RT-PCR. Each experiment was repeated three times using RNA prepared from three biological samples with similar results.

RT-PCR analysis was performed to study the expression of LT responsive *Arabidopsis CBFs* and *COR* genes. The results showed that in *TaVRN2* transgenic plants, the expression level of *AtCBFs* and *AtCOR* genes tested are higher than the controls plants (Fig. 7 & Fig. S1). These results suggest that over expressing *TaVRN2* in transgenic plants mimic low temperature molecular response.

## Discussion

Molecular characterization of *VRN2* in hexaploid wheat demonstrates sequence variations between the homologous *TaVRN2* genes. *TaVRN-A2* and *TaVRN-B2* exhibit marked sequence variation and higher level of transcript accumulation. This could be explained by the fact that the A, B and D genomes evolved at different rates [32]. This variation also supports the idea that various events of sequence changes have occurred in the evolutionary history of wheat homeologous genomes that diverged about 5.0–6.9 million years ago [33]. Indeed, since the polyploidization event of wheat, significant sequence changes have occurred that might cause mutation or lost of *TaVRN2* in modern (spring) wheat. Alignment of the CCT domain of TaVRN-A2 and TaVRN-B2 shows a point mutation at position 35 of the CCT domain, where a tryptophan (W) substitutes for an arginine (R) amino acid. Mutation within this domain results in that *TaVRN-A2* encodes a non-functional protein as shown previously [21]. This observation may explain why *TaVRN-A2* is expressed at a very low level to the extent that we could not clone it from our cDNA libraries. It is not known, however, if the gene is expressed into non-functional proteins or is not translated at all.

The *TaVRN2* genes are also regulated by photoperiod, dehydration, wounding, heat shock and ABA stresses. This indicates that *TaVRN2*, besides having a major role in regulating vernalization and photoperiod responses, might integrate signals from other environmental stresses to execute its functions during wheat adaptability and development. This is supported by the presence of several putative regulatory *cis*-elements in its promoter region. Down regulation of the flowering repressor *TaVRN2* under stress conditions could be used as a strategy to promote flowering and ensure the survival of the species. Flowering time could also be determine by combinatorial responses as in the case of *TaVRN1* and *TaVRT2*, which are regulated by the combination of both low temperature and day length [13], [26], [34]. However, the biological significance of the regulation of *TaVRN2* by several stresses requires further investigation.

Transgenic *35S::TaVRN2* lines showed a late-flowering phenotype indicating that the repressive effects of TaVRN2 are recognized by certain proteins of *Arabidopsis* flowering pathways. In these lines, the expression level *AtFVE*, *AtFT* and *AtSOC1* was either slightly increased or similar after 7 days of growth under LD conditions. The increase was more evident after 14 days of growth. However, at both time points, the level of *AtFLC* in transgenic plants was 4 times higher than in control plants. This suggests that despite the observed increased of *AtFVE*, *AtFT* and *AtSOC1*, the *AtFLC* level still high enough to counteract the action of these flowering inducer genes and delays flowering time in plants overexpressing *TaVRN2* (Figure 5A).

A model where AtCO is recruited by another direct DNA-binding and interacts with AtFLC in *35S::AtCO 35S::AtFLC* transgenic plants to repress AtSOC1 factor was proposed [35]. TaVRN2 protein, a zinc finger transcription factor like AtCO, could bind the CCAAT sequence in the *AtSOC1* promoter and regulates its transcription. This hypothesis remains to be confirmed. Taken together, our data indicates that the late flowering of the 35S::*TaVRN2* plants was associated with a complex pattern of expression of the major flowering control *Arabidopsis* genes *FCA*, *FLC*, *FT*, *FVE* and *SOC1*. This suggests that heterologous expression of *TaVRN2* in *Arabidopsis* can also delay flowering by modulating several floral inductive pathways. In cereals, it has been reported that low temperature and daylength flowering-response pathways are integrated to control expression of VRN3 (the AtFT orthologue) and that might occur through regulation of VRN2 [36], [37]. In contrast, our data did not show a repression of *AtFT* by *TaVRN2* over-expression, suggesting the possibility of TaVRN2 may act through another pathway in *Arabidopsis*. Overexpressing *TaVRN2* in mutants of these flowering genes will provide the answer to the exact molecular and cellular function of *TaVRN2* gene in *Arabidopsis*.

It is known that the length of the vegetative phase in vernalization sensitive plants is associated with the development of freezing tolerance [2]. Plants lose their capacity to cold acclimate once the vernalization saturation point is achieved. Thus extending the vegetative phase in *Arabidopsis* by overexpressing *TaVRN2* should mimic low temperature response. Our data confirms this hypothesis and showed that the delay in the transition to the reproductive phase is associated with enhanced freezing tolerance. This increase in tolerance is partly due to the induction of *AtCBFs* and *AtCORs* genes. *TaVRN2* could have a dual role by regulating flowering time and response to cold as proposed for *AtFVE* [38]. However, the exact cellular functions of *TaVRN2* in *Arabidopsis* require further investigation.

Flowering time in plants plays a major role in plant adaptation, particularly in temperate region. Premature flowering initiation has a negative effect on productivity. Thus, identifying key genes involved in regulating this important trait will facilitate the development of molecular markers that will be used in breeding programs. It will be of interest to extend the vegetative phase in cold sensitive species to avoid flowering under unfavorable condition. On the other hand, accelerating flowering could be useful to shorter the life cycle for certain species and thus maximize the use of land resources and increase yield.

## Supporting Information

Table S1 List of primers used in this study.
(0.15 MB PDF)

Sequences S1 TaVRN-A2 and TaVRN-B2 open reading frame sequences with specific primers of ZCCT1 highlighted in grey and probe highlighted in yellow. Genomic sequences of TaVRN-A2 copy and two promoters from spring wheat cv Manitou and one promoter from winter wheat cv Norstar.
(0.03 MB DOC)

Figure S1 Effect of TaVRN-B2 over-expression on the accumulation of cold-regulated transcripts. A) Transcript level of CBFs genes. Control (35S::GUS) and transgenic 35S::TaVRN-B2 plants (line 1) grown under long day conditions and exposed to 4°C for 3 and 6 hours. B) Transcript level of CORs genes. Control (35S::GUS) and transgenic 35S::TaVRN-B2 plants (line 1) grown under long day conditions at 4°C for 1 and 2 weeks. Panels A and B are scanned for densitometry measurement. Relative expression is normalized in relation to the expression of CBF-2 of transgenic 35S::TaVRN-B2 exposed to 4°C for 3 hours (panel A) and to the expression of COR 15a of transgenic 35S::TaVRN-B2 exposed to 4°C for one week (panel B).
(1.17 MB TIF)
