# Supplementary material for: Heterologous Expression of Wheat VERNALIZATION 2 (TaVRN2) Gene in Arabidopsis Delays Flowering and Enhances Freezing Tolerance
Source: PLoS One. 2010 Jan 13;5(1):e8690. doi: 10.1371/journal.pone.0008690 (PMC2805711; doi:10.1371/journal.pone.0008690)
Supplement: Table S1 — List of primers used in this study. (0.15 MB PDF) [file pone.0008690.s001.pdf]

Table S1: List of primers used in this study

**Table S1. Primers used for the different experiments on the characterization of the wheat *TaVRN2* gene.**	
**Primers used to clone genes and promoters of *TaVRN2*.**	
TaVRN2-F	5'-ATGTCCATGTCATGCGGTTTGT-3'
TaVRN2-R	5'-TTACCGGAACCATCCGAGGTGAA 3'
TaVRN2-F2	5'-GTTAAGCTTGGGGGAGAAGAGTCC-3'
TaVRN2-R2	5'-GGTGGTGGTGGTTGCCTTG-3'
**Primers used for the generation of construct used to overexpress *TaVRN2* in *Arabidopsis thaliana*.** The restriction enzyme cutting sites used for cloning are underlined.	
TaVRN2-F3	5'-CGGATCCTATGTCCATGTCATGCGGTTTGT-3'
TaVRN-R3	5'-AGGAATTCTTTACCGGAACCATCCGAGGTGA-3'
**Primers used for qRT-PCR analyses of transcripts expressed in wheat.**	
TaVRN-B2/D2-F4	5'-CCGACACATGGCTCACCTAGTG-3'
TaVRN-B2/D2-R4	5'-TTGCTTCATTGCTAATAGTGTTTGT-3'
TaVRN-A2-F5	5'-CGACCCATGGCTCACCTAGT-3'
TaVRN-A2-R5	5'-TTGCTTCATTGCTAATAGTGTTGGT-3'
TaVRN2-PROBE	5'-GGCAAGAGCCCACATCGTGCCATTT-3'
**Primers used for RT-PCR analyses of transcripts expressed in *Arabidopsis thaliana* and in wheat.**	
AtCBF1-F	*5'-TGGAAGCTATTTATACACCGGAAC-3'*
AtCBF1-R	*5'-GTACAAAAATGGAAACGACTATCGAAT-3'*
AtCBF2-F	*5'-ACCTTGGTGGAGGCTATTTATACG-3'*
AtCBF2-R	*5'-CATTTGCATTTGACAACAACTTTTACC-3'*
AtCBF3-F	*5'-CAGAGCGAAAATGCGTTTTATATGCA-3'*
AtCBF3-R	*5'-TAATTTACACTCGTTTCTCAGTTTTACA-3'*
AtCOR6.6-F	*5'-GTGTTAACTTCGTGAAGGACAAG-3'*
AtCOR6.6-R	*5'-CAAACGTAGTACATCTAAAGGGAGA-3'*
AtCOR15a-F	*5'-GATACATTGGGTAAAGAAGCTGAGA-3'*
AtCOR15a-R	*5'-CGGTGACTGTGGATACCATATCTT-3'*
AtCOR78-F	*5'-GTTGAAGAGTCTCCACAATCACTT-3'*
AtCOR78-R	*5'-AATCCAATGATTTTACCCACTTTAGAC-3'*
AtFCA-F	5'- AATGTACCTGGACCGAGCATACCT-3'
AtFCA-R	5'-CTGCTGAACTTGTTGTGGTTGTTG-3'
AtFLC-F	5'-CGGTTGAAATCAAAATCCAAAACA-3'
AtFLC-R	5'-CACACGAATAAGGTACAAAGTTCATC-3'
AtFVE-F	5'-ATGCAGATACTAACTGGGCACCAA-3'
AtFVE-R	5'-AATCTGTCCCAATCGTTGTGATGT-3'
AtSOC1-F	5'-ACCATAGATCGTTATCTGAGGCAT-3'
AtSOC1-R	5'-GAAGAACAAGGTAACCCAATGAAC-3'
AtFT-F	5'-TAGTAAGCAGAGTTGTTGGAGACG-3'
AtFT-R	5'-GGGAAGGCCGAGATTGTAGAT-3'
Actin2-F	5'-TCAGATGCCCAGAAGTGTGTT-3'
Actin2-R	5'-CCGTACAGATCCTTCCTGATAT-3'
TaVRN2 forward	5'-GGCGGCCGACACATGGCTCA-3'
TaVRN2 reverse	5'-TGGGCAGGCCCCACCATCATC-3'
TaVRT2 forward	5'-GTGGCCGTTGCCGAAGCTGAAAAT-3'
TaVRT2 reverse	5'-CGCGCCATGCAAATGGAGACATAAAACGA-3'
TaVRN1 forward	5'-GCTGAAGGGCTTCCAGCCCATATAAG-3'
TaVRN1 reverse	5'-TACATGGTAAATTGAGCCCAGCTGGG-3'
18S forward	5'-AGTTAAAAAGCTCGTAGTTGGACCT-3'
18S reverse	5'-GTTTATGGTTGAGACTAGGACGGTA-3'
