# Supplementary material for: Heterologous Expression of Wheat VERNALIZATION 2 (TaVRN2) Gene in Arabidopsis Delays Flowering and Enhances Freezing Tolerance
Source: PLoS One. 2010 Jan 13;5(1):e8690. doi: 10.1371/journal.pone.0008690 (PMC2805711; doi:10.1371/journal.pone.0008690)
Supplement: Sequences S1 — TaVRN-A2 and TaVRN-B2 open reading frame sequences with specific primers of ZCCT1 highlighted in grey and probe highlighted in yellow. Genomic sequences of TaVRN-A2 copy and two promoters from spring wheat cv Manitou and one promoter from winter wheat cv Norstar. (0.03 MB DOC) [file pone.0008690.s002.doc]

**Sequences S1:** *TaVRN-A2 and TaVRN-B2* open reading frame sequences with specific primers of ZCCT1 highlighted in grey and probe highlighted in yellow.

Genomic sequences of *TaVRN-A2* copy and two promoters from spring wheat cv Manitou and one promoter from winter wheat cv Norstar.

**>*TaVRN-A2*, 621pb amplified fron genomic DNA (cv Norstar and cv Manitou), the sequence in obtained after delating the intron.**

ATGTCCATGTCATGCGGTTTGTGCGGCGCTAACAACTGCCCGCGCCTCATGGTCTCGCCCATTCATCATCGTCATCACCATCATCAGGAGCACCAGCTGCGTGAGCACCAGTTCTTCGCCCAAGGCAACCACCACCACCACCACCCAGTGCCACTGCCGCCAGCCAACTTCGACCACAGCAGAACATGGACCACACCATTTCATGAAACAGCAGCTGCAGGGAACAGCAGCAGGCTCACGCTGGAGGTGGGCGCAGGCGGCCGACCCATGGCTCACCTAGTGCAGCCACCGGCAAGAGCCCACATCGTGCCATTTTACGGAGGTGCATTCACCAACACTATTAGCAATGAAGCAATCATGACTATTGACACAGAGATGATGGTGGGGCCTGCCCATTATCCCACAATGCAGGAGAGAGCAGCGAAGGTGATGAGGTATAGGGAGAAGAGGAAGAGGCGGCGCTATGACAAGCAAATCCGATATGAGTCCAGAAAAGCTTACGCTGAGCTTCGGCCATGGGTCAACGGCCGCTTTGTCAAGGTACCCGAAGCCATGGCGTCGCCATCATCTCCAGCTTCGCCCTATGATCCTAGTAAACTTCACCTCGGATGGTTCCGGTAA

**>*TaVRN-B2*_ORF_642pb amplified from the winter wheat cold acclimated (cv Norstar) cDNA libraries**

ATGTCCATGTCATGCGGTTTGTGCGGCGCCAACAACTGCTCGCGCCTCATGGTCTCGCCCATTCATCATCATCATCACCATCATCAGGAGCACCAGCTGCGTGAGCACCAGTTCTTCGCCCAAGGCAACCACCACCACCACCACCATGGCGCGGCAGTAGACCACCCAGTGCCACCGCCGCCAGCCAACTTCGACCACCGCAGAACATGGACTACACCATTTCATGAAACAGCAGCTGCAGGGAACAGCAGCAGGCTCACACTGGAGGTGGGCGCAGGCGGCCGACACATGGCTCACCTAGTGCAGCCACCGGCAAGAGCCCACATCGTGCCATTTTATGGAGGTGCATTCACAAACACTATTAGCAATGAAGCAATCATGACTATTGACACAGAGATGATGGTGGGGCCTGCCCATTATCCCACAATGCAGGAGAGAGCAGCGAAGGTGATGAGGTATAGGGAGAAGAGGAAGAGGCGGCGCTATGACAAGCAAATCCGATACGAGTCCAGAAAAGCTTACGCTGAGCTCCGGCCACGGGTAAACGGCCGCTTCGTCAAGGTACCCGAAGCCATGGCGTCGCCATCATCTCCAGCTTTGCCCTATGGTCCTAGTAAACTTCACCTCGGATGGTTCCGGTAA

***TaVRN2*_genomic_NORSTAR_copy_A_1871pb**

ATGTCCATGTCATGCGGTTTGTGCGGCGCTAACAACTGCCCGCGCCTCATGGTCTCGCCCATTCATCATCGTCATCACCATCATCAGGAGCACCAGCTGCGTGAGCACCAGTTCTTCGCCCAAGGCAACCACCACCACCACCACCCAGTGCCACTGCCGCCAGCCAACTTCGACCACAGCAGAACATGGACCACACCATTTCATGAAACAGCAGCTGCAGGGAACAGCAGCAGGCTCACGCTGGAGGTGGGCGCAGGCGGCCGACCCATGGCTCACCTAGTGCAGCCACCGGCAAGAGCCCACATCGTAAGTAGTAGTACCGCTTAATTGTTTCATCTCTTGCCGATGGATGCGTCCCTGGCTTCCTCCTTAAAAATCCCCACCTAATTTATGTCCATCTATACCCACTACAAAAAAATAGCACCATGTAACCATCTCATATATCTGTCACATAATTCTGTTAATGTACGCTGCTCAATTGTTCTCCTGAAAAAGATATGCGGGAATGGATCTTGATATTCTTTAATTTTCTATGGAGGCATATATAGAGTTTGTGTTTTGTATTAGTTGATGCAGAATTGTATGGGTTGTCAAATCATCAGTCATACATATAAACTTATTTCATTTTATTTGACCAACAACAAGGTAATCAGTCATACATGCATACTGAAAATTTGACTTGTGTTCAATAACTAACCAACTCGACCGGCACAGCTGGGGGAAGACTTTAATCAAGCTGCTAGCTAGAGCTTAATAATATAACATATCTCTTTATGGGATCAAGCAATACATATGCGCTCAATTCTCAACTTGTCAATATCTATCTGGAGTCCACACTTTATGGTAATTAATTGACAAAGTTTTGTGAAATGGACAATATACATACTGGATCGATGCACCCTTTTTCTCATTTTATGTGGTCATTATGAATTTGATTGTTATTTAATATTTCAATTTTATCTTGAGCTAGTTTTGCAAGTCTGTAGCTCATATATAACTGATACTACTCCCCACGATAGCTTGCGTAGTGGCCGGGTGATCGATCTACCGAGTTCATAAAACTGATCGAGATCGGGTCCAAAAAAGAACAAACCCATACAAAATGGAAAGAAGATCCTTGTTTAGTTAGTTTGCATCAGAAAATTGCCTAATTAGTTACTTGCTATCAATCTTTTGAACATGGCATGTTCACCCCAAACGGACCCAGATCACAATTATTGATGAAGTTACGCCTTTTAAAAACTCATAAAACTGTACATACATGTACAGGGCTACACACATGTACATAATACACCTAATTAAAACGTATATTCGTAGACCAATTGTTTTGGACGGTGCGCATCTTTGAAAAAAAAATGCCAGAGGAGTTGTTAGCTTCCACTGTCCAGAAATAGAATAGTTACAATCAAGTGCATCTCTGAATGAAAATGGATCATTTTCTAGTTAATTAGAGACCAATTAGATACTTCATAAACAGGGGAGTATCAAGTACGTATCTGCTACCCTAAGAAAGTACATAACTGCGATCTTATGATTATTTTCCTCTTGATGTTCAGGTGCCATTTTACGGAGGTGCATTCACCAACACTATTAGCAATGAAGCAATCATGACTATTGACACAGAGATGATGGTGGGGCCTGCCCATTATCCCACAATGCAGGAGAGAGCAGCGAAGGTGATGAGGTATAGGGAGAAGAGGAAGAGGCGGCGCTATGACAAGCAAATCCGATATGAGTCCAGAAAAGCTTACGCTGAGCTTCGGCCATGGGTCAACGGCCGCTTTGTCAAGGTACCCGAAGCCATGGCGTCGCCATCATCTCCAGCTTCGCCCTATGATCCTAGTAAACTTCACCTCGGATGGTTCCGGTAA

***TaVRN2*_genomic_MANITOU_ copy_A_1872pb**

ATGTCCATGTCATGCGGTTTGTGCGGCGCCAACAACTGCCCGCGCCTCATGGTCTCGCCCATTCATCATCGTCATCACCATCATCAGGAGCACCAGCTGCGTCAGCACCAGTTCTTCGCCCAAGGCAACCACCACCACCACCACCCAGTGCCACTGCCGCCAGCCAACTTCGACCATAGCAGAACATGGACCACACCATTTCATGAAACAGCAGCTGCAGGGAACAGCAGCAGGCTCACGCTGGAGGTGGGCGCAGGCGGCCGACCCATGGCTCACCTAGTGCAGCCACCGGCAAGAGCCCACATCGTAAGTAGTAGTACCGCTTAATTGTTTCATCTCTTGCCGATGGATGCGTCCCTGGCTTCCTCCTTAAAAATCCCCACCTAATTTATGTCCATCTATACCCACTACAAAAAAATAGCACCATGTAACCATCTCATATATCTGTCACATAATTCTGTTAATGTACGCTGCTCAATTGTTCTCCTGAAAAAGATATGCGGGAATGGATCTTGATATTCTTTAATTTTCTATGGAGGCATATATAGAGTTTGTGTTTTGTATTAGTTGATGCAGAATTGTATGGGTTGTCAAATCATCAGTCATACATATAAACTTATTTCATTTTATTTGACCAACAACAAGGTAATCAGTCATACATGCATACTGAAAATTTGACTTGTGTTCAATAACTAACCAACTCGACCGGCACAGCTGGGGGAAGACTTTAATCAAGCTGCTAGCTAGAGCTTAATAATATAACATATCTCTTTATGGGATCAAGCAATACATATGCGCTCAATTCTCAACTTGTCAATATCTATCTGGAGTCCACACTTTATGGTAATTAATTGACAAAGTTTTGTGAAATGGACAATATACATACTGGATCGATGCACCCTTTTTCTCATTTTATGTGGTCATTATGAATTTGATTGTTATTTAGTATTTCAATTTTATCTTGAGCTAGTTTTGCAAGTCTGTAGCTCATATATAACTGATACTACTCCCCACGATAGCTTGCGTAGTGGCCGGGTGATCGATCTACCGAGTTCATAAAACTGATCGAGATCGGGTCCAAAAAAGAACAAACCCATACAAAATGGAAAGAAGATCCTTGTTTAGTTAGTTTGCATCAGAAAAATTGCCTAATTAGTTACTTGGCTATCAATCTTTTGAACATGGCATGTTCACCCCAAACGGACTCAGATCACAATTATTGATGAAGTTACGCCTTTTAAAAACTCATAAAACTGTACATACATGTACAGGGCTACACACATGTACATAATACACCTAATTAAAACGTATATTCGTAGACCAATTGTTTTGGACGGTGCACATCTTTGAAAAAAAATGCCAGAGGAGTTGTTAGCTTCCACTGTCCAGAAATAGAATAGTTACAATCAAGTGCATCTCTGAATGAAAATGGATCATTTTCTAGTTAATTAGAGACCAATTAGATACTTCATAAACAGGGGAGTATCAAGTACGTATCTGCTACCCTAAGAAAGTACATAACTGCGATCTTATGATTATTTTCCTCTTGATGTTCAGGTGCCATTTTACGGAGGTGCATTCACCAACACTATTAGCAATGAAGCAATCATGACTATTGACACAGAGATGATGGTGGGGCCTGCCCATTATCCCACAATGCAGGAGAGAGCAGCGAAGGTGATGAGGTATAGGGAGAAGAGGAAGAGGCGGCGCTATGACAAGCAAATCAGATACGAGTCCAGAAAAGCTTACGCTGAGCTTCGGCCACGGGTCAACGGCTGCTTTGTCAAGGTACCCGAAGCCATGGCGTCGCCATCATCTCCAGCTTCGCCCTATGATCCTAGTAAACTTCACCTCGGATGGTTCCGGTAA

>**Promoter TaVRN2 copy 2 from Manitou 760 pb**

GGTTAAGCTTGGGGGAGAAGAGTCCTCGCTGGTTCTCATTCTAGAGTTTAGTTTTCCATGCCCATGATAATAGCATGGATGCCCCATGACGAAAATTGTTTCACAGCTGGTAGTACTTTTCTATTTTAGTATTGGCATGGTTTCCATTTTGTTGTTTTTGTCTCCCTCGGACTTTTGTGTTAGCATCTCCTTTTTGTTTTGACGCTGACCAAAAAAAGCTACACAAATATCTAGCAGTGGCCTTGTGTGGACATAAGATCATGTGGGGGATTCCCAGCAAGCAAGGTCTGCATGGCTCCGGCTCCTCCGCGTAAGAAAGAAAGAAATCAACGATGGATCGAGGGATCATATCTATTCCGACCCACTCATTAGTTGAGCAATATTTTGATAGTTGCCATATCGAATATTTTTTCTGGCCTGAGAGCTCACGGCTGCCTATATGCAGTGCATGTGAGAGAGACACAGTACGGCCCTAGCTACTACTACAAGTACCTTGGTAGTTACTGGTACTCATAACTGCCTCTTCTTCTTCCTCGACATCTCTCCTCCTCGGCCCCTCCACGCACCAGACCACAGCAGAAAAAACAAACAAGCAAGCAAACCTTGGAGCTAGCTAGCAGT**ATG**TCCATGTCATGCGGTTTGTGCGGCGCCAACAACTGCCCGCGCCTCATGGTCTCGCCCATTCATCATCGTCATCACCATCATCAGGAGCACCAGCTGCGTCAGCACCAGTTCTTCGCCCAAGGCAACCACCACCACC

>**Promoter TaVRN2 copy 1 from Manitou 763 pb**

GGTTAAGCTTGGGGGAGAAGAGTCCTCGCTGGTTCTCATTTTACAGTTTAGTTTTCCATGCCCATGATATAGTATGGATGGCCCATGACAAAAGTTGTTTCACAACTGGTAGTACGTTTCTATTTGAGGTTCTATTTTAGTATTGACATGGTTTCCATTTTGTTGTGTTTGTCTCCCTCGGACCTTTGTGTTAGCATCTCCTTTTTGTTTGACACTGACCAAAAAAAAGTTACACAAATATCTAGCAGTGGGCTTGTGTGGACATAAGATCATGTGGGGGATTCCCGGCAAGCAAGGTCTGCATGGCTCCGACTCCTCCACGTAAAAAAGAAAGAAATCAACGATCGATCGAGGGACCATATCTATTCCGACCCACTCATTAGTTGGGTCTATTTGATTTGATCCATTGTATTTTGCTAATTCCCATATCGAATCTTTTTTCTGGCCTTGGAGCTCACTGCTGCTTATATGCGGTGCATGTGAGAGAGAGACGCAGTACAGCCCTAGCTGCTAGTACAACTGCCTCTTCTTCTTTCTCGACATCTCTCCTCCTCGGATCCTCCACGCACCAGACCACACCAGAAAAAACAAACAAGCAAGCAAACCTTGGAGCTAGCTAGCAGT**ATG**TCCATGTCATGCGATTTGTGCGGCGCCAACAACTGCTCGCGCCTCATGGTCTCGCCCATTCATCATCATCATCACCATCATCAGGAGCACCAGCTGCGTGAGCACCAGTTCTTCGCCCAAGGCAACCACCACCACC

>**Promoter TaVRN2 copy from Norstar 763 pb**

GGTTAAGCTTGGGGGAGAAGAGTCCTCGCTGGTTCTCATTTTACAGTTTAGTTTTCCATGCCCATGATATAGTATGGATGGCCCATGACAAAAGTTGTTTCACAACTGGTAGTACGTTTCTATTTGAGGTTCTATTTTAGTATTGACATGGTTTCCATTTTGTTGTGTTTGTCTCCCTCGGACCTTTGTGTTAGCATCTCCTTTTTGTTTGACACTGACCAAAAAAAAGTTACACAAATATCTAGCAGTGGGCTTGTGTGGACATAAGATCATGTGGGGGATTCCCGGCAAGCAAGGTCTGCATGGCTCCGACTCCTCCACGTAAAAAAGAAAGAAATCAACGATCGATCGAGGGACCATATCTATTCCGACCCACTCATTAGTTGGGTCTATTTGATTTGATCCATTGTATTTTGCTAATTCCCATATCGAATCTTTTTTCTGGCCTTGGAGCTCACTGCTGCTTATATGCGGTGCATGTGAGAGAGAGACGCAGTACAGCCCTAGCTGCTAGTACAACTGCCTCTTCTTCTTTCTCGACATCTCTCCTCCTCGGATCCTCCACGCACCAGACCACACCAGAAAAAACAAACAAGCAAGCAAACCTTGGAGCTAGCTAGCAGT**ATG**TCCATGTCATGCGATTTGTGCGGCGCCAACAACTGCTCGCGCCTCATGGTCTCGCCCATTCATCATCATCATCACCATCATCAGGAGCACCAGCTGCGTGAGCACCAGTTCTTCGCCCAAGGCAACCACCACCACC
