# Supplementary figures and images for: Heterologous Expression of Wheat VERNALIZATION 2 (TaVRN2) Gene in Arabidopsis Delays Flowering and Enhances Freezing Tolerance
Source: PLoS One. 2010 Jan 13;5(1):e8690. doi: 10.1371/journal.pone.0008690 (PMC2805711; doi:10.1371/journal.pone.0008690)

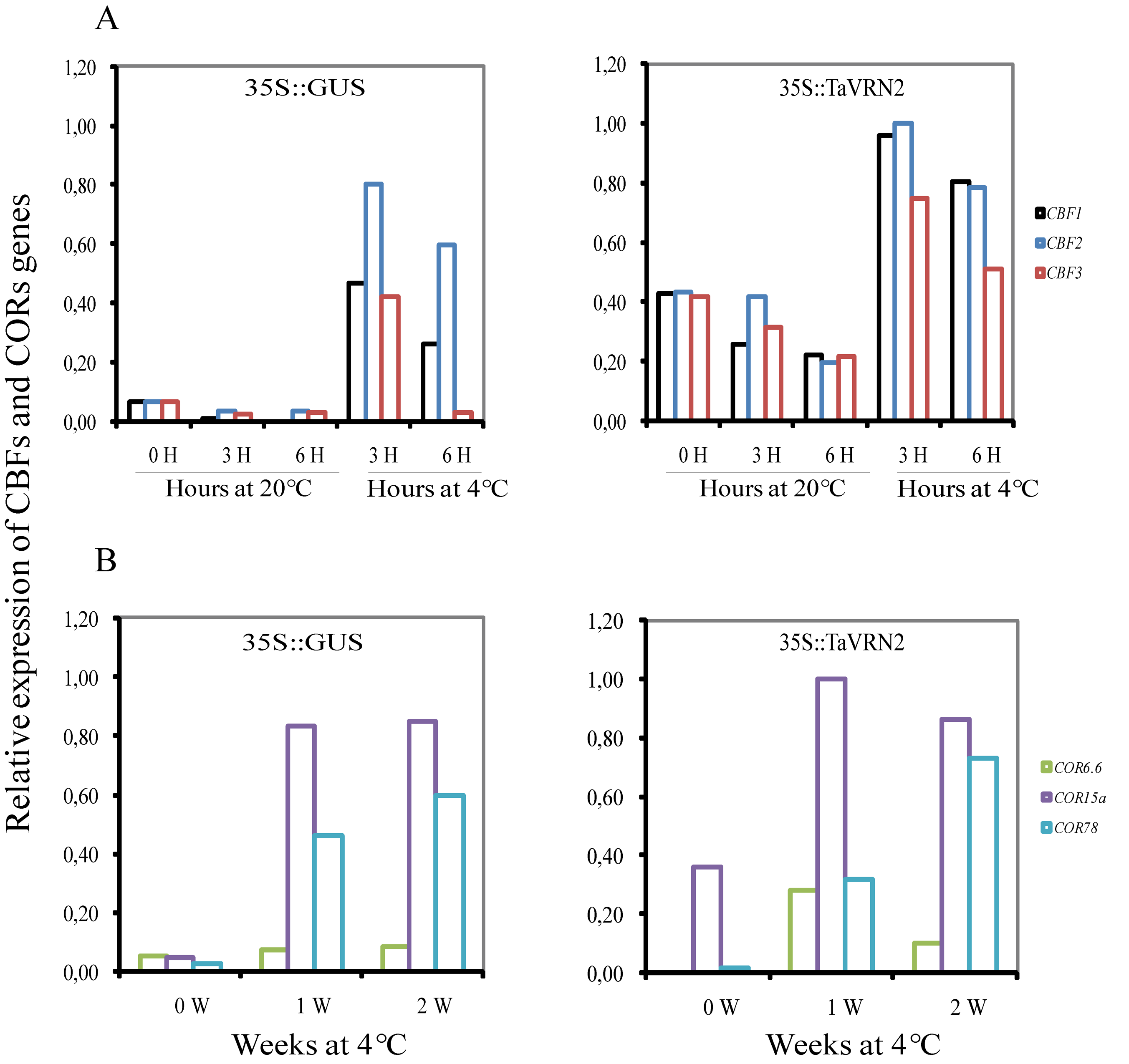

Supplement: Figure S1 — Effect of TaVRN-B2 over-expression on the accumulation of cold-regulated transcripts. A) Transcript level of CBFs genes. Control (35S::GUS) and transgenic 35S::TaVRN-B2 plants (line 1) grown under long day conditions and exposed to 4°C for 3 and 6 hours. B) Transcript level of CORs genes. Control (35S::GUS) and transgenic 35S::TaVRN-B2 plants (line 1) grown under long day conditions at 4°C for 1 and 2 weeks. Panels A and B are scanned for densitometry measurement. Relative expression is normalized in relation to the expression of CBF-2 of transgenic 35S::TaVRN-B2 exposed to 4°C for 3 hours (panel A) and to the expression of COR 15a of transgenic 35S::TaVRN-B2 exposed to 4°C for one week (panel B). (1.17 MB TIF) [file pone.0008690.s003.tif]
